# Self-Care as a Method to Cope With Suffering and Death: A Participatory Action-Research Aimed at Quality Improvement

**DOI:** 10.3389/fpsyg.2022.769702

**Published:** 2022-02-21

**Authors:** Loredana Buonaccorso, Silvia Tanzi, Simona Sacchi, Sara Alquati, Elisabetta Bertocchi, Cristina Autelitano, Eleonora Taberna, Gianfranco Martucci

**Affiliations:** ^1^Psycho-Oncology Unit, Azienda USL – IRCCS di Reggio Emilia, Reggio Emilia, Italy; ^2^Palliative Care Unit, Azienda USL – IRCCS di Reggio Emilia, Reggio Emilia, Italy; ^3^Azienda Sanitaria dell’Alto Adige, Comprensorio Sanitario di Merano, Merano, Italy; ^4^Local Network of Palliative Care, Azienda USL Modena, Modena, Italy

**Keywords:** self-care, self-awareness, compassionate presence, palliative care (MeSH), continuous education, action-research

## Abstract

**Introduction:**

Palliative care is an emotionally and spiritually high-demanding setting of care. The literature reports on the main issues in order to implement self-care, but there are no models for the organization of the training course. We described the structure of training on self-care and its effects for a Hospital Palliative Care Unit.

**Method:**

We used action-research training experience based mostly on qualitative data. Thematic analysis of data on open-ended questions, researcher’s field notes, oral and written feedback from the trainer and the participants on training outcomes and satisfaction questionnaires were used.

**Results:**

Four major themes emerged: (1) “Professional role and personal feelings”; (2) “Inside and outside the team”; (3) “Do I listen to my emotions in the care relationship?”; (4) “Death: theirs vs. mine.” According to participants’ point of view and researchers’ observations, the training course resulted in ameliorative adjustments of the program; improved skills in self-awareness of own’s emotions and sharing of perceived emotional burden; practicing “compassionate presence” with patients; shared language to address previously uncharted aspects of coping; allowing for continuity of the skills learned; translation of the language learned into daily clinical practices through specific facilitation; a structured staff’s support system for emotional experiences.

**Discussion:**

Self-care is an important enabler for the care of others. The core of our intervention was to encourage a meta-perspective in which the trainees developed greater perspicacity pertaining to their professional role in the working alliance and also recognizing the contribution of their personal emotions to impasse experienced with patients.

## Introduction

Palliative care (PC) is an emotionally and spiritually high-demanding setting of care. Professionals working in the landscape of death are frequently exposed to existential issues, psychological challenges, and emotional distress associated with care at the end of life (Cole and Carlin, 2009; Sinclair, 2011). The risks of working in this context are well documented, e.g., burnout, compassion fatigue, and poor quality of care (Cole and Carlin, 2009; Sinclair, 2011; Peters et al., 2012). Self-care is an important enabler for the care of others (Kearney et al., 2009). The healthcare professionals (HCPs) must take care of their own health and well-being to support their competence in caring for patients (Mount et al., 2007; Kearney et al., 2009; Zulman et al., 2020).

The training in self-reflection of one’s emotional experience and its meanings associated with suffering, death and dying asks the HCPs to focus on their own resources and coping mechanisms (Meier et al., 2001; Kearney et al., 2009). It might result in better outcomes for both HCPs and the patients and their families (Meier et al., 2001; Kearney et al., 2009; Childers and Arnold, 2019). Research on the training of self-care, self-awareness of one’s emotions, spirituality and inner life of the HCPs, according to available resources and local context on these topics, is still needed.

Many international documents reported that the PC training in self-care skills might be relevant for the HCPs’ well-being and for the patients (Jünger et al., 2010; Jünger and Payne, 2011; Società Italiana di Cure Palliative, 2016; Brighton et al., 2019; Joint Royal Colleges of Physicians Training Board, 2020).

The Italian core curricula was developed from Italian Association of PC containing indications on “self-care and awareness” (Società Italiana di Cure Palliative, 2016). It suggests that the HCPs should have access to a space in which to reflect on one’s emotional experiences that are associated with the assistance of suffering and dying patients. It emphasized the relevance of having a high level of awareness of one own’s emotions, prejudices, projections, beliefs, and level of stress. It also suggests developing these skills with active educational strategies as role playing, and creating occasions of team discussions and opportunities for personal spiritual reflection during work time.

The European Association of PC white paper on spiritual care education (Best et al., 2020) suggests developing reflective capacity of staff: “Self-awareness can help the healthcare practitioner to avoid being distracted by their own fears, prejudices and restraints and attend to the patient.” Furthermore, Puchalski et al. (2019) suggested that learners should reflect on their own spirituality in practicing compassionate presence, on their professional call to serve, and on their spiritual beliefs and self-care practices. Clinicians can apply a compassionate presence, reflective listening, referral to dignity therapy, forgiveness therapy, and self-care spiritual practice (e.g., meditation and yoga) through case-based presentation.

Nonetheless, there is no one-size-fits-all solution as indicated by the scientific literature. All the previously referenced literature report on the main issues to be addressed in order to implement self-care and self-awareness of emotions, but the organization of the training courses (e.g., How many hours and days? What themes? What professional figures should be involved? Which setting: indoor or outdoor?) is an aspect in which there are no univocal models. Considering that inner life is a highly cultural-sensitive topic, it might be important in every context to coherently develop its own answer.

Therefore, we considered it imperative to describe a training experience on self-care to identify the specific characteristics of the topics covered, the number of hours identified, and the professionals involved.

## Aim

We described the structure of a training on self-care and its effects for a Hospital Palliative Care Unit.

## Methods

### Setting

This study was conducted with a Specialized Palliative Care Service (SPCS) at an Italian hospital, in the context of continuous medical education, from September 2018 to April 2019. The SPCS is a specialized unit with no designated hospital beds. It was established in April 2013 with a remit of specialist in-hospital consultations and in a clinic for oncological and non-oncological outpatients and their family members. It is continuously involved in its own medical education and training courses to improve spiritual and psycho-social care, as well as courses to solve ethical dilemma (De Panfilis et al., 2020).

The SPCS includes three senior PC physicians and two advanced practice nurses. A psychologist psychotherapist expert in palliative care is also available for the care of patients and family members with severe psychological distress.

### Subjects and Recruitment

We used a purposive sample by enrolling the entire staff of the SPCS in the training that consisted of three PC physicians (ST, SS, and SA), one resident doctor (ET), and two nurses (CA and EB).

### Research Design

The research design was a participatory action-research of a training experience (Smith et al., 2010). An action-research approach in PC has been suggested as a possible way to both address an existing problem in a specific context and produce useful scientific knowledge by different authors (Hockley and Froggatt, 2006; Froggatt and Hockley, 2011; Hockley and Stacpoole, 2014). Our research was a “professionalizing type” action research, according to Hockley and Froggatt (2006), and the educational purpose was to “enhance professional control and individual ability to control work situation” (Smith et al., 2010; Hockley and Stacpoole, 2014). In this specific type of study design, the research was meant to be the response to a problem “defined by professional group” and “related to a behavior of practitioners,” as it was considered “successful” relatively to a contested definition of success (Smith et al., 2010; Hockley and Stacpoole, 2014). “Observation” as a research methodology can be “participative” or “non-participative,” depending on the choice and the possibilities of the researcher of taking part in the actions that he/she is also observing, which usually results in the production of field notes. Field notes were taken at a short distance from the events and iteratively enriched with additional reflections and insights of the researcher, so that he/she can get to a “thick description” of the observed phenomena. A participatory project usually allows for the elaboration of *ad hoc* instruments used to investigate preferences and choices of the participants.

**FIGURE 1:**
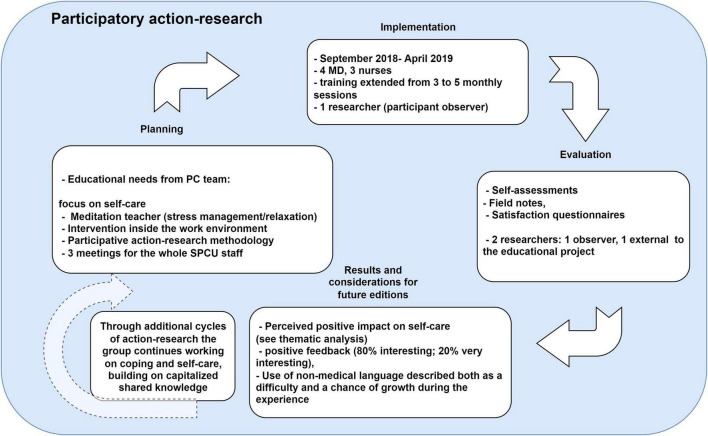
The different phases of action research adapted to our study.

In our study, field notes have been taken in different moments during both participative and non-participative observation by one researcher (LB). A second researcher (GM) facilitated the participative evaluation of the events with trainer and trainees (see Figure 1).

During the usual assessment of educational needs, aimed at the programming of the continuous education plan for the following months, the need to develop a training program on the topic of personal perspective and relation of the professionals with death, and the impact of this on their work life, emerged. LB who works as a psychologist psychotherapist with the SPCS, identified the trainer based on the routine educational needs assessment, which takes place during dedicated meetings where every participant can propose topics for the future training. The researcher (LB) maintained the role of note-taking and documentation during and after the training (see Figure 1).

Self-care and self-awareness of one’s own emotions were selected as the main focuses of the training. Consequently, the principal study question of the action-research was as follows:

“*How can we develop an educational answer to our need of better coping with the strong emotional distress due to our constant relation with death in the professional environment of a palliative care unit?”*

A secondary study question, according to the action-research methodology was as follows:

“*Which useful lessons can we draw from our educational experience in the development of similar initiatives?”*

### Intervention

In accordance with internal procedures, the intervention was endorsed by the competent body of the Hospital (CME Scientific Committee): 30.4 CME were recognized. A trainer with a background in the field of non-technical skills related to the self-awareness of one’s own emotions was chosen. She is a meditation teacher with specific expertise in stress management and relaxation. The training was provided in three sessions for a total of 16 h (September 2018–December 2018). After three meetings, the trainees expressed a need to extend the training by an additional two meetings for a total of 6 h (February 2019–April 2019). They expressed the difficulties of understanding a language more focused on self-awareness of personal emotions.

The meetings started from significant clinical cases brought in by the participants with the focus on diseases, suffering, listening to one’s emotions starting from psycho-physical relaxation, and self-reflection on one’s response mechanism to suffering (see Table 1 for curricular details for each session). A training particularly based on a constructivist approach that “values the cultivation of trainee self-reflection, relational awareness, and creativity in envisioning alternative therapeutic strategies,” was used (Novack et al., 1997; Neimeyer et al., 2016). It supported the trainees to take the lead in analyzing and deconstructing their own in-session behaviors from a stance of self-awareness rather than self-criticism. Albeit demanding, the recursive and reflexive nature of the questions that scaffolded the training seemed to consistently help trainees shift to a meta-perspective in which they developed greater perspicacity for their role in the working alliance without seldom recognition of the contribution of their personal preoccupations or insecurities to impasse experienced in the care relationship (Neimeyer et al., 2016).

**TABLE 1:** Curricular details for each session of the intervention.

Date	Topics	Methodology	Time
September 2018	- Self-awareness in the care relationship - Listening to one’s emotions - Process the emotions within a caring relationship; understand the pain, suffering, and the issues related to the search of meaning - Meditation: Relaxing the physical body through breathing	Interactive lesson, brainstorming, sharing of clinical cases and experiences	4 h
October 2018	- Disease and suffering from the point of view of yoga and meditation - Relationship between the patient’s suffering and our emotions - Listening to oneself in the care relationship	Outdoor meeting Individual and group work	8 h
November 2018	- Meditation: Relaxing the physical body through breathing - Sharing of clinical cases	Individual and group work	4 h
February 2019	- Meditation: Relaxing the physical body through breathing - Death and Life from the point of view of the healthcare professionals	Brainstorming	3 h
April 2019	- Meditation techniques: relaxation of the body, breathing, and the visualization of colors - Use of breath during the communication with the patients and in the care relationship	Individual and group work	3 h

During the intervention and after its conclusion, the researchers (LB and GM) conducted sessions of training analysis and reflection on the impact, starting from the collected materials (answers to evaluation questions and field notes) to the subsequent participative meetings aimed at evaluating and reflecting on the results with trainers and participants (see Figure 1). In particular, as specified in Figure 1, LB supervised the self-assessment of the perceived educational needs of the group, and all the training was based on an inductive approach, where the participants were invited to share their experiences and personal emotions relevant in their daily activity as a starting point for the session. In the following year, LB facilitated a reflective attitude on what emerged, taking notes on spontaneous reflections on the training and using open ended questions to keep track of the considerations of the team on the contents and the strategies that they learnt in the training. To increase the trustworthiness of the analysis and the reflexivity of the group, GM examined all the data as an independent, external researcher and facilitated two sessions of reflections on the draft version results in the draft version.

### Data Collection and Analysis

The researchers (LB and GM) administered both a pre-and post-training with an assessment test of open-ended questions. It consisted of 13 questions anonymously investigating the relationship of death and the ability of listening to one’s own needs and emotions, based on the literature from these topics (Novack et al., 1997) (see Table 2 for the contents of the questions).

**TABLE 2:** Pre- and post-training open-ended questions assessment test (Novack et al., 1997).

	Questions
1	Do you find pleasure in your work?
2	If so, in what context? - Otherwise, explain your lack of pleasure
3	What is the quality of your emotions and your thoughts? What words do you express and use during your work?
4	What words would you like to say? What words do you not want to or unable to express?
5	What do you take home with you from your work? (i.e., what is left inside after you leave?)
6	What is left of your job in your life?
7	What would you like to change about your job?
8	In what ways, if so, would you like your colleagues to behave?
9	Why did you choose this job? What do you expect from your job?
10	Can you describe a positive word and a negative word that describe your colleagues?
11	How do you listen to yourself? How do you relate to other people?
12	How do you deal with disease and death?
13	Could you write a single word that describes death for you?

The researchers (LB and GM) conducted a thematic qualitative analysis (Elliott et al., 1999; Braun and Clarke, 2006; Gale et al., 2013; Hsieh and Shannon, 2016) of all materials (pre- and post-tests integrated with the field notes).

To generate initial codes, they independently labeled the texts and met to discuss them after. As the first step, the creation of categories and themes was developed with a “paper and pencil” approach. After themes emerged in the final shared list, researchers sorted and ordered data in charts using ATLAS qualitative analysis software. Subsequently, labels were combined to search for themes and sub-themes, comparing possible differences in the researchers’ points of view. Both had assigned tags to the text through a recursive and iterative process of deduction and induction until they were able to select the most relevant themes and sub-themes (Elliott et al., 1999; Hsieh and Shannon, 2016).

Afterward, the themes were reviewed and refined to assure their internal coherence. As such, a consensus among the researchers was reached in defining and naming the themes. Researchers identified as many themes as possible to accurately represent the content of the complete data set, and the findings were described through a thematic description, in order to explain the meaning of the most predominant and relevant themes (Hsieh and Shannon, 2016).

The resulting core themes were discussed with all trainees (Braun and Clarke, 2006; Gale et al., 2013) in a reflective session after the conclusion of the formal training with the purpose of gaining their opinion on the results and adding relevant information to the results.

### Ethical Considerations

Because the study addressed educational practices and quality improvement, the hospital’s Internal regulation on the competent body of the Hospital (CME Scientific Committee) did not require specific informed consent procedures. Nevertheless, all participant information was handled as confidential, and informed participant’s consent was verbally gathered throughout. As the participation in the training implied involvement in the study, teacher made sure that participants were fully aware of this.

## Results

### Thematic Analysis

Four major themes emerged from the notes taken by the two researchers (LB and GM) and the pre-and post-tests: (1) “Professional role and personal feelings”; (2) “Inside and outside the team”; (3) “Do I listen to my emotions in the care relationship?”; (4) “Death: theirs vs. mine (see Table 3 for the major theme exemplars pre-test and post-test).”

**TABLE 3:** Major Theme Exemplars pre-test and post-test.

Themes	Verbatim of the trainees pre-training	Verbatim of the trainees post-training
*“Professional role and personal feelings”*	“*I would like to say a word of closeness, but my professional role and the fear of being less competent are stopping me.*” (P1) “*Sometimes I would like to say something not only about the work, but also about a personal daily life (…), words that, however, refer me to an inability.*” (P2) “*I seem unable to say “in words” that I am there for that person, who is unique and beautiful, to make her feel precious. This thing makes me feel inefficient*.” (P3)	“*I find pleasure when I feel effective in what I do, if I am aware of what I do. I find pleasure when I am myself*” (P3) *“I can almost always express what I think, and I know how to be silent when this is worth more than a word.*” (P6)
*“In and outside the team”*	*“I wish my colleagues would take more moments to share their emotions because I think they can relate to me.”* (P2) *“I would like my colleagues to have more patience with colleagues from other Service”* (P3)	“*I would like the colleagues of the other Services to see more of the beauty of what I do, so they would be more cooperative. However. it’s not bad.”* (P1) *“I would like my colleagues from the other Services to listen more to themselves and to others.”* (P6) “*As for “non-working” relationships, I would like us to take less for granted, help each other more, and also share how we feel.”* (P3) “*I would like my colleagues to be cooperative and non-judgmental to better share our skills and knowledge with colleagues from other departments*” (P5)
*“Do I listen to me? Not always”*	“*I listen to myself at times but not always in depth.*” (P1) “*I listen to the physical symptoms because I somatize everything*.” (P4)	“*I listen to myself by realizing my physical sensations and understanding my reactions.*” (P1) “*I think a lot to keep something from what I live.*” (P3) “*I listen to myself all the time and try to be in tune with myself.*” (P6) “*I try to use irony to help relieve myself of tensions.*” (P5)
*“The death: the others and not mine”*	“*I try to realize that it is a part of life, even if it is very difficult to be aware of it; I struggle to think of myself in the path of illness”*(P2) “*My greatest fear is physical suffering; with death my relationship is calmer.*” (P5)	“*I relate to the disease by facing it, keeping it in mind with death and consequently giving great importance to life.*” (P1) “*Death is there, but it is not seen. We would not even want to see it if we could; patients have far more resources than we think we have for ourselves. They amaze us and teach us.*” (P6)

(1) “*Professional role and personal feelings*”

During the pre-test, the participants expressed the need to say “words of proximity” to patients. However, they also expressed a fear of being perceived as less competent inside and outside of the professional role. The fear that the patient could leave also emerged, and this emotional component was perceived as inadequate.

“*Sometimes I would like to say something not only about the work, but also about a personal daily life (…), words that, however, refer me to an inability*” (P2).

According to the post-test, the discrepancy between professional role and personal identity seemed to decrease. Furthermore, a greater awareness of *presence* in the care relationship and the recognition of its *quality* emerged.

“*I find pleasure when I feel effective in what I do, if I am aware of what I do. I find pleasure when I am myself*” (P3).

At the end of the training, the trainees used phrases centered more on the feeling of an increased “lightness” regarding their emotional burden, based on the research’s file notes during the team meetings (LB). During the meetings the trainees focused more on their emotions by recognizing and expressing them as well as talking about their difficulties in the relationship.

(2) “*Inside and outside the team”*

According to the pre-test, a distinction between the team colleagues and colleagues from the other services of the hospital developed due to the participants’ expectations of greater collaboration from their colleagues from other units.

*“I would like my colleagues to have more patience with colleagues from other Service”* (P3).

At the end of the course, a greater understanding toward colleagues outside the team was reported in the evaluation forms and team meetings.

“*I would like the colleagues of the other Services to see more of the beauty of what I do, so they would be more cooperative. However, it’s not bad”* (P1).

The trainees also reported more interest in the emotional experience of colleagues from their own and other services, according to the field notes of the researcher (LB).

(3) “*Do I listen my own emotions in the care relationship?”*

During the pre-test, sporadic listening was reported, regarding one’s own emotions.

Some participants reported developing the habit of listening more to themselves during and after the medical visits, starting from physical symptoms and others carving out an individual inner space.

“*I listen to myself at times but not always in depth*” (P1).

At the end of the training, there was a greater emphasis in listening to oneself and motivation to share one’s emotional states with colleagues.

“*I listen to myself by realizing my physical sensations and understanding my reactions*” (P1).

(4) “*Death: theirs* vs. *mine”*

According to the pre-test, death was recognized as present in the care relationship, even when it is not named.

“*I try to realize that it is part of life, even if it is very difficult to be aware of it; I struggle to think of myself in the path of illness”* (P2).

At the end of the training, the participants recognized the death of the patients as well as their own, as seen from the thematic analysis.

“*I relate to the disease by facing it, keeping it in mind with death and consequently giving great importance to life*” (P1).

In reference to the research field notes (LB), the importance of dealing with death by talking about dignity, in particular in reference to the Model of Chochinov (2002), and part of the team’s background was revealed.

Table 4 shows the results of the satisfaction questionnaires after the intervention. In general, the trainees found the themes of the training interesting (80% interesting; 20% very interesting), however, from the field notes difficulties became evident regarding the language used which, having been not technical, because the trainer does not work in the hospital setting.

**TABLE 4:** Results of the satisfaction questionnaires.

Question	% of respondents for each value of the scale reported in brackets. Total number of respondents: 6
*Were the training objectives presented clearly?* (*In a 1–6 scale*)	20% (4) 60% (5) 20% (6)
*Have the aims been achieved?* (*In a 1–6 scale*)	40% (4) 40% (5) 20% (6)
*Is what I learned consistent with the skill requirements of my professional activities? (In a 1–6 scale)*	20% (4) 60% (5) 20% (6)
*What have I learned is concretely applicable in my clinical practice?* (*In a 1–6 scale*)	40% (4) 40% (5) 20% (6)
*Was the teaching method effective?* (*In a 1–6 scale*)	40% (4) 40% (5) 20% (6)
*Were the themes interesting?* (*In a 1–6 scale*)	80% (5) 20% (6)

## Discussion

Self-care is an important enabler to the care of others. In our study, the meditation teacher was chosen with the aim to improve self-awareness of emotions in the care relationship and to foster self-care, starting from body relaxation and use of meditation techniques.

Our study identified a methodology in the action-research to improve a training program with satisfaction and qualitative evidence of the impact on the palliative practice. The participants identified oral and written feedback from the trainer and the researchers (LB and GM) during and after the training, as a method to improve the course contents. The researcher’s field notes taken during and after the training have been deemed as useful to customize the teaching (see Figure 1). Due to the increasing need in field of self-care, the action-research may be an effective way of developing programs to have a real impact on a professional’s practice in accordance with available resources.

HCPs discussed meaningful experiences with patients, listening to themselves in a broader concept of “learning to learn” (Bateson, 1997) from self-awareness of own emotions rather than self-criticism, as suggested by a constructivist and self-compassion approach (Petrocchi et al., 2014; Neimeyer et al., 2016; Neff et al., 2020). At the end of the training, there was a greater emphasis in listening to oneself and motivation to share one’s emotional states with colleagues, supported by a self-compassion approach. This approach involves responding kindly to one’s own suffering and failures, rather than neglecting one’s well-being or engaging in judgment and self-criticism (Petrocchi et al., 2014; Neff et al., 2020), according to the field notes of the researcher (LB). The oral and written feedback from participants revealed important changes developed during the team meetings, such as greater attention was given to one’s emotional experience during the discussion of clinical cases. Phrases like “*This patient is…” and “These families are…*” were changed to “*My difficulty was…,” “I felt that way,”* and *“I ask the team for help*,” as reported in the research’s files notes (LB). Some authors underlined that effective self-care practice involves self-awareness, self-compassion, and the implementation of a variety of strategies across inner life domains (Mills et al., 2020; Moroni et al., 2020). Greater awareness of the ability to cultivate positive emotions such as self-compassion should be promote as a part of self-care practice, as reported in a systematic integrative review on self-compassion in hospice and PC (Garcia et al., 2021).

At the end of the training, the participants also reported more interest in the emotional experience of colleagues from their own and other services. A qualitative study that explored the meaning and practice of self-care in PC HCPs, reported that self-compassion was considered essential for self-care and related to compassion for others, as our study suggested (Mills et al., 2018). In particular, treating oneself with kindness, awareness of common humanity, and avoiding over identification with emotions can contribute to the personal well-being of hospice and PC HCPs (Garcia et al., 2021). The trainees actively applied these principles that also emerged from the Dignity Model (Buonaccorso et al., 2021; Tanzi and Buonaccorso, 2021). The lesson learned from the training course applied to the pandemic was that having a compassionate presence during the short visits to isolated COVID-19 patients at the Infectious Disease Unit helped to restore an increased perception of dignity and humanity (Tanzi et al., 2020; Tanzi and Buonaccorso, 2021). Solitary death in the extraordinary emergency that HCPs have experienced has required increased skills and closeness to these patients, in terms of compassion and conscious presence (Moroni et al., 2020). The compassionate support, self-care, and quality of life are also important concerns for the HCPs (Adams et al., 2020; Mills et al., 2020). Prioritizing self-care by developing a plan is an effective strategy that can be supported by a specific intervention as Self-Care Matters, a free resource available online^1^. It is drawn from contemporary research to shed light on understanding, practicing, and planning self-care through the voice of experienced clinicians (Palliative Care Australia, 2020).

Individual self-care plans in combination with Staff Supportive initiatives are indicated as practice to prioritize in such emergencies (Mills et al., 2015, 2018; Sansó et al., 2015). As reported in a cross-sectional online survey of PC Spanish professionals, the cultivation of inner life for better professional quality of life and compassionate care, and its repercussion on professionals’ wellbeing, take place across sex, age, and controlling for important work variables, such as work overload or workload control. When compared to these traditional organizational variables, self-compassion and coping with death have stronger effects on professional quality of life, which emphasizes the importance of properly cultivating an inner life in HCPs to provide compassionate care (Galiana et al., 2021).

Through the training, the our team began to reflect on a self-care plan as a team that considered personal needs in the context of PC. For the incoming year two training on “spirituality” and “compassion and self-compassion” were organized with spiritual assistants and psychotherapists who work in hospice and PC to reflect on their own spirituality and compassionate presence as HCPs.

### Useful Tips for Future Editions

Reflective sessions with the participants highlighted some possible improvements of the initial program, useful for future editions of similar initiatives in their context or in similar environments. In our experience, it was useful to keep an open discussion with the participant on the right number of sessions. In fact, while three meetings were initially planned, the group decided to extend the training to five meetings total, to give participants the time to learn meditative techniques within specific PC context and to get more familiar with a new language related to non-medical disciplines. As a result, in our model we developed a solution of five meetings of 4 h, monthly.

Furthermore, one session was conducted outdoor, to facilitate the learning of physical body relaxation and meditation techniques.

The core of our intervention was to encourage a meta-perspective in which the trainees developed greater perspicacity pertaining to their professional role in the working alliance and also recognizing the contribution of their personal emotions to impasse experienced with patients. In our experience, the trainees reported the importance of identifying a trainer who carries out at least part of his activity within a context of patient care. This, from our experience, would make it easier to use a “common” language. The limit of identifying trainers outside the CP setting could be linked to the fact of using techniques that belong to other traditions (Buddhist, Tibetan…) without, however, integrating them with Western culture and in the context of PC. The oral and written feedback from participants revealed the importance of having a dedicated operator who already works with the team and who is able to translate the language learned during the training into daily clinical practices. In our study, this work of continuing the learned contents was facilitated during the following team meetings in which the operator (LB) participated in her usual role as supporting psychologist.

Coping with death and awareness are important predictors of quality of life, being positively related to Compassion Satisfaction (Galiana et al., 2021; Garcia et al., 2021). In the increasingly needed field of self-awareness, a more participatory format, like the action-research design, may be an effective way of improving programs to have a real impact on professionals’ practice in accordance with available resources and local context’s needs. This methodology allows to better tailor the educational experience, especially considering that the language used to speak about self-awareness can be difficult to develop.

### Limitations

Regarding limitations, we highlight that our sample size of six participants was small. This was due to the type of intervention in that only the working staff of the interested unit was necessary to be involved to build a more tailored training experience. However, our study is a qualitative research/data analysis that is not intended to generalize the results. In this way, we wanted to propose an experience to contribute to advancing knowledge in an area of increasing importance in PC—self-care as a method to cope with suffering and death.

Furthermore, the SPCS had already participated in other courses on bioethics, psychological skills, and spirituality. Therefore, the training was aimed at HCPs previously trained on similar topics. Finally, the presence of a competent figure as a psychologist who gives continuity to the contents after the course and helps the team to translate them into practice might be a great facilitator for learning, but local resources do not always allow the continuous presence of a psychologist.

While this qualitative reporting of an impactful experience may help in developing similar and useful experiences, we acknowledge that only different and more quantitative types of researchers could help to understand the optimal elements and combined impact of staff support and self-care and the method that these can be best implemented in normal circumstances and also adjusted to respond to situations of heightened stress or complexity (Sansó et al., 2015; Adams et al., 2020).

## Data Availability Statement

The original contributions presented in the study are included in the article/supplementary material, further inquiries can be directed to the corresponding author/s.

## Ethics Statement

Ethical approval was not provided for this study on human participants because the study addressed educational practices and quality improvement, consequently the hospital’s Internal regulation on the competent body of the Hospital (CME Scientific Committee) did not require specific informed consent procedures. Ethical review and approval was not required for this study on human participants in accordance with the local legislation and institutional requirements. Nevertheless, all participant information was handled as confidential, and informed participant’s consent was verbally gathered throughout. As the participation in the training implied involvement in the study, teacher made sure that participants were fully aware of this. Written informed consent for participation was not required for this study in accordance with the national legislation and the institutional requirements.

## Author Contributions

LB analyzed the literature, contributed to the conception of the study, the draft of the manuscript, and critical revision with relevant theoretical content, supporting each member of the team and approved the final version to be submitted and agreed to be accountable for all aspects of the work ensuring that questions related to the accuracy or integrity of any part of it are appropriately investigated and resolved. ST analyzed the literature, contributed to the draft of the manuscript and to its critical revision, together with SS, SA, CA, EB, and ET took part to the training and provided iterative feedback, decisions on focus and structure of the training, evaluation of perceived outcomes, and the role of co-researchers as typical of a participatory action research. GM analyzed the literature and gave a methodological contribution, in particular on qualitative methods and contributed to the conception of the study, to the draft of the manuscript, and to its critical revision and approved the final version to be submitted and agreed to be accountable for all aspects of the work ensuring that questions related to the accuracy or integrity of any part of it are appropriately investigated and resolved. All authors read and approved the final manuscript.

## Conflict of Interest

The authors declare that the research was conducted in the absence of any commercial or financial relationships that could be construed as a potential conflict of interest.

## Publisher’s Note

All claims expressed in this article are solely those of the authors and do not necessarily represent those of their affiliated organizations, or those of the publisher, the editors and the reviewers. Any product that may be evaluated in this article, or claim that may be made by its manufacturer, is not guaranteed or endorsed by the publisher.

## Footnotes

1
https://palliativecare.org.au/resources/self-care-matters
